# The impact of menstrual cycle phase and symptoms on sleep, recovery, and stress in elite female basketball athletes: a longitudinal study

**DOI:** 10.3389/fphys.2025.1663657

**Published:** 2025-09-23

**Authors:** Lisa Kullik, Eduard Isenmann, Jan Schalla, Michael Kellmann, Sarah Jakowski

**Affiliations:** ^1^ Department of Sport Psychology, Faculty of Sport Science, Ruhr University Bochum, Bochum, Germany; ^2^ Department of Fitness and Health, IST-University of Applied Sciences, Düsseldorf, Germany; ^3^ School of Human Movement and Nutrition Sciences, The University of Queensland, Saint Lucia, QLD, Australia

**Keywords:** menstrual cycle, sleep, recovery, basketball, elite athletes

## Abstract

**Aim:**

This study aimed to examine the influence of menstrual cycle phases and symptom burden on sleep quality and recovery-stress states in elite female basketball players.

**Methods:**

Initially, twelve elite athletes participated in a 3-month observational study, which included psychometric screening using validated questionnaires and daily monitoring of menstrual symptoms, subjective sleep quality, sleep parameters, and recovery-stress states. The final analysis included eight athletes (26.75 ± 5.63 years, 178.62 ± 7.48 cm, 68.94 ± 7.13 kg, average cycle length of 29.00 ± 1.20 days, menstruation duration of 5.75 ± 0.71 days). In addition to self-reported data, objective menstrual cycle parameters were collected using the Ava fertility tracker. To verify cycle regularity, salivary hormone samples were collected twice weekly. Data analysis was conducted using linear mixed modeling to account for repeated measures and intra-individual variation.

**Results:**

Across both approaches, menstrual cycle phases showed only limited and inconsistent associations with sleep and recovery-stress states. In contrast, higher daily symptom burden and greater overall symptom frequency were consistently associated with poorer sleep quality, reduced recovery, and elevated stress. Additionally, sleep behavior significantly influenced both sleep and recovery outcomes.

**Conclusion:**

Symptom burden appears to be a more relevant factor than menstrual phase in determining sleep and recovery-stress states in elite female athletes. These findings support individualized monitoring approaches that include menstrual symptoms tracking. Psychoeducation on sleep hygiene and menstrual health should be integrated into elite sports environments to strengthen athlete well-being. Overall, the study highlights the importance of multidimensional, athlete-centered strategies that combine behavioral, hormonal, and symptom-based data to optimize performance and recovery.

## 1 Introduction

Sleep is an essential component of training, recovery, performance, and overall well-being in athletes (Kellmann et al., 2018; Kölling et al., 2019). Regarding the high physical and psychological demands from frequent exposure to intense training and competition, elite athletes require greater amounts of restorative sleep to maintain adequate recovery and peak performance (Andrade et al., 2019; Lastella et al., 2014; Sargent et al., 2014). Due to intense training loads, frequent travel, and irregular competition schedules, elite athletes are particularly vulnerable to sleep disturbances (Cook and Charest, 2023; Walsh et al., 2021). However, optimal performance in sports can only be achieved and maintained if a balanced relationship exists between stress and recovery (Kellmann et al., 2018). Despite athletes themselves reporting that sleep is a primary factor contributing to fatigue and underperformance (Venter, 2014), they often show unfavorable sleep quality (Andrade et al., 2019), sleep durations below 7 hours (Roberts et al., 2019; Vlahoyiannis et al., 2021) as well as worse wake after sleep onset, and sleep efficiency compared to non-exercising controls (Leeder et al., 2012).

While these findings highlight the general relevance of sleep in athletic populations, sport-specific research offers more targeted insights. Evidence from collegiate basketball players has shown that extending nightly sleep to 10 h over several weeks led to significant improvements in sprint times, shooting accuracy, and mental well-being (Mah et al., 2011). Moreover, higher subjective sleep quality was reported as associated with improved offensive ratings and player efficacy (Fox et al., 2021). A study involving Division I female basketball athletes revealed that improved recovery-related parameters were closely associated with enhanced performance and a reduction in injuries (Senbel et al., 2025). Players showed improvements in sleep quality and resting heart rate, accompanied by enhanced recovery and reduced stress during the more successful season (Senbel et al., 2025). Similar findings were acknowledged in collegiate soccer players, where greater sleep duration and quality had increasingly protective effects on perceived exertion, soreness, and psychological stress throughout the season, highlighting the long-term importance of sleep for athlete well-being and recovery (Long et al., 2022).

Despite the fundamental role of sleep and athletic recovery, female athletes are underrepresented in research on sleep and recovery (Power et al., 2024). Although women’s sports is gaining visibility and participation is rising, research on female-specific physiological and psychological needs remains limited (McNulty et al., 2024a). Only a small proportion of sport science studies include women as primary participants, and even fewer systematically account for menstrual cycle phases (Vogel et al., 2023). The resulting lack of robust, sex-specific data hinders the development of evidence-based recommendations, particularly in team sport contexts where individualized load and recovery management is challenging due to logistical and structural barriers. In high-performance settings, hormonal effects on sleep remain underexplored, highlighting the value of menstrual cycle tracking for self-awareness, dysfunction detection, and symptom management (Carmichael et al., 2025). Awareness of how hormonal status, training load, and sleep interact is increasing, but solid evidence is still limited (Power et al., 2024).

Understanding the menstrual cycle is crucial for comprehending the intricate relationship between hormonal status, training, and sleep. Fluctuations in estrogen and progesterone affect systems relevant to athletic performance, including neuromuscular function, thermoregulation, metabolism, motivation, and sleep (Bernstein and Behringer, 2023; Constantini et al., 2005; McNulty et al., 2020). These hormonal changes contribute to individual variability in fatigue, recovery, and training responses (Elliott-Sale et al., 2021). The early follicular phase, when hormone levels are lowest, is often associated with favorable training conditions, such as improved fatigue resistance and lower perceived exertion (Lee et al., 2024; Sims et al., 2021). In the late follicular phase and around ovulation, rising estrogen may enhance neuromuscular function and endurance (Sung et al., 2014). Studies in professional basketball players show better performance and recovery during the follicular phase compared to the luteal phase (Arenas-Pareja et al., 2023; Gasperi et al., 2023). However, increasing estradiol levels may also lead to increased joint laxity and a higher risk of injury due to altered neuromuscular control (Julian et al., 2017; Oosthuyse and Bosch, 2010). The luteal phase is characterized by elevated levels of estrogen and progesterone, which can lead to reduced recovery capacity, increased perceived exertion, and greater vulnerability to stressors (Draper et al., 2018; Lee et al., 2024; Sims et al., 2021).

These physiological factors may also affect recovery-stress states (De Martin Topranin et al., 2023; Scott et al., 2024) and sleep, with longer wake time, lighter sleep, and lower efficiency reported particularly in the mid-luteal phase (Taylor et al., 2024). However, beyond phase-specific hormonal fluctuations, symptom-related influences are frequently neglected in menstrual cycle research (Bruinvels et al., 2022). Still, these symptoms are highly prevalent (Taim et al., 2023) and perceived by many athletes to negatively affect training and competition performance, primarily due to fatigue, pain, and reduced endurance (Armour et al., 2020). Multiple studies have reported fatigue, mood changes, and cramps as the most reported complaints (Bruinvels et al., 2021; Kullik et al., 2024; Nolan et al., 2023). A higher frequency of menstrual symptoms was significantly associated with poorer sleep quality and more unfavorable sleep behaviors (Kullik et al., 2024). Additionally, Halson et al. (2024) demonstrated in elite soccer players that the number of daily symptoms, rather than the specific cycle day, was significantly associated with increased sleep duration and more wake after sleep onset, suggesting that symptoms disrupt sleep continuity. Similarly, Brown et al. (2025) reported that subjective sleep quality decreased on days with higher menstrual symptom load, and that poorer sleep was associated with greater perceived stress and worse mood states.

Importantly, athletes themselves view the menstrual cycle as a very underrated part of performance, training, and stress (Jørgensen et al., 2025). These insights underscore the importance of acknowledging and integrating menstrual health into athlete care and training planning. The number of symptoms experienced was strongly associated with this perceived disruption (Brown and Duffield, 2024). This was acknowledged in a sample of handball athletes, where more than 30% reported that menstrual cycle-related symptoms frequently affect their performance (Osborne et al., 2025). In a recent study on elite female rugby players, Hayward et al. (2024) reported that nearly 88% of professional female rugby players in the United Kingdom perceived their menstrual cycle as negatively affecting their training or competitive performance, with common symptoms including fatigue, decreased strength, impaired focus, and emotional instability.

Even when studies from different sports report similar findings about the impact and athletes’ views on the importance, a one-size-fits-all approach is unlikely to be effective, due to the distinct characteristics and environments of each sport (Carmichael et al., 2025). Ultimately, the current state of research underscores the urgent need to integrate menstrual cycle monitoring into comprehensive athlete recovery strategies, particularly in team sports such as basketball, where seasonal fatigue accumulates and performance output is highly variable (Edwards et al., 2018). Thus, this study aims to investigate the relationship between menstrual cycle phases, menstruation-related complaints (such as symptoms or menstrual bleeding intensity), and sleep, recovery, and stress in elite female basketball players. It is assumed that athletes show decreased sleep parameters during the luteal phase (H1). A similar effect is hypothesized for recovery-stress states (H2). Furthermore, it is assumed that menstrual symptoms are associated with impaired sleep parameters (H3) and decreased recovery-stress states (H4).

## 2 Materials and methods

### 2.1 Sample

Healthy female athletes from two basketball teams in North Rhine-Westphalia, Germany (divisions 1 and 2), were recruited for this observational study. According to the New Paradigm for Participant Training and Performance Classification (McKay et al., 2022), the athletes were classified as Tier 3 (Highly Trained/National) and Tier 4 (Elite/International). Twelve athletes initially agreed to participate. Ultimately, eight with a natural menstrual cycle were included in the analysis. All athletes gave their written informed consent prior to participation. The local ethics committee granted ethical approval for the study (Reference: EKS V 17/2021). The study procedures adhered to the ethical standards outlined in the Declaration of Helsinki as well as the ethical standards of Ruhr University Bochum. The descriptive sample description is presented in Table 1.

**TABLE 1:** Descriptive sample description (n = 8).

	Mean ± SD	Min	Max	Median
Age, years	26.75 ± 5.63	17.00	31.00	29.00
Height, cm	178.62 ± 7.48	170.00	190.00	177.50
Weight, kg	68.94 ± 7.13	61.00	80.00	67.50
BMI	21.56 ± 19.88	19.88	22.84	21.57
Cycle length, days	29.00 ± 1.20	28.00	31.00	28.50
Menstruation length, days	5.75 ± 0.71	5.00	7.00	6.00
MSi score	24.75 ± 12.53	4	40	26
ASBQ score	41.62 ± 5.68	30	46	43.5

BMI, Body-Mass-Index; MSi, Menstrual Symptom index; ASBQ, athlete sleep behavior questionnaire.

### 2.2 Procedure

**FIGURE 1:**
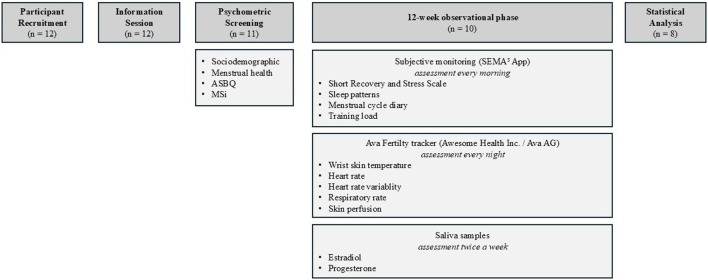
Schematic study design. Note: ASBQ = Athlete Sleep Behavior Questionnaire; MSi = Menstrual Symptom index.

Before the study began, participants attended an information session that provided an overview of the theoretical background and organizational procedures. They were also informed about the objective instruments and how to use them. The study design consisted of psychometric screening followed by a 12-week observational phase from September to December 2022 during the regular basketball season of both divisions. The complete study design is schematically illustrated in Figure 1.

### 2.3 Measurements

#### 2.3.1 Psychometric screening

Before the start of the observational phase, participants completed a screening questionnaire that included questions about their athletic background, sociodemographic data, and information about the menstrual cycle, including regularity, disturbances, contraception, and the Menstrual Symptom Index (MSi; Bruinvels et al., 2021), to assess symptom frequency systematically. The index consists of 18 symptoms, with their frequency rated on a four-point Likert scale ranging from 0 (*never*) to 3 (*often*). The MSi score was calculated as the sum of all item scores, resulting in a total score ranging from 0 to 54. The Athlete Sleep Behavior Questionnaire (ASBQ; Driller et al., 2018) was included to consider athlete-specific sleep patterns. It provides a practical tool for professionals in psychology and sports science to evaluate the sleep behavior of elite athletes. It assesses the frequency of specific sleep-related behaviors using a five-point Likert scale ranging from 1 (*never)* to 5 (*always*). A global score is calculated by summing all item responses, resulting in a total score ranging from 0 to 90. Higher scores indicate poorer sleep behavior. Scores of 36 or below reflect good sleep behavior, whereas scores above 42 suggest poor sleep behavior.

#### 2.3.2 Monitoring

The SEMA^3^ mobile application (O’Brien et al., 2024) was used for daily monitoring. Participants were instructed to complete the protocol within 30 min after awakening. The Short Recovery and Stress Scale (SRSS; Kellmann and Kölling, 2019; Kellmann and Kölling, 2020) measured the current recovery-stress state. The SRSS was developed as a shortened version of the Acute Recovery and Stress Scale. It captures recovery and stress across emotional, mental, physical, and general domains. The items are rated on a seven-point Likert scale from 0 (*does not apply at all*) to 6 (*fully applies*) and consist of Physical Performance Capability, Mental Performance Capability, Emotional Balance, and Overall Recovery for the recovery dimension, and Muscular Stress, Lack of Activation, Negative Emotional State, and Overall Stress for the stress dimension. Higher scores reflect higher levels of recovery or stress within the respective domains. Given the substantial interindividual variability, SRSS scores should be interpreted in the context of repeated assessments, taking into account intraindividual changes over time. The scale shows robust construct validity and a high sensitivity to change (Kellmann and Kölling, 2020).

The protocol also monitored participants’ sleep-wake patterns. The athletes reported their bedtime, the duration of time they spent in bed before attempting to fall asleep, sleep onset latency, wake after sleep onset, and wake-up time. Subjective sleep quality was assessed by the question “*How did you sleep last night?*” (1 = *bad*; 2 = *rather bad*; 3 = *medium*; 4 = *rather good*; 5 = *good*) and restfulness of sleep by the question “*How restful was your sleep*” (1 = *not restful at all*; 5 = *very restful*). Moreover, the athletes documented a menstrual cycle diary in addition to the objective assessment. They reported the presence of menstruation, the specific day of the menstruation, intensity of menstrual bleeding measured on a five-point Likert scale (1 = *very light*; 2 = *light*; 3 = *medium*; 4 = *heavy*; 5 = *very heavy*), and which symptoms they experienced, based on the symptom list of the MSi (0 = *no*; 1 = *yes*). To document the training load, the participant retrospectively documented the previous day’s practice, including the number of practice sessions and practice duration. They were also asked to report on their perceived exertion, measured by the Rating of Perceived Exertion (RPE; Foster et al., 2001). To quantify the internal load, the perceived training load (sRPE) was calculated by multiplying the reported RPE by the corresponding session duration in minutes. Moreover, it was documented whether the athletes were injured and how it affected their practice participation (*I did not practice*; *I practiced with limitations*; *I practiced normally*).

#### 2.3.3 Ava fertility tracker

The Ava fertility tracker (Awesome Health Inc., USA, formerly Ava AG, Switzerland) was used to determine cycle phases using physiological data. The bracelet was worn at night during sleep. The device utilizes photoplethysmography (PPG) technology to measure wrist skin temperature, heart rate variability ratio (HRV ratio), respiratory rate, and skin perfusion. The HRV ratio is defined as the ratio between the low-frequency and high-frequency components of heart rate variability, serving as an indicator of physiological stress. This measure differs from overall HRV and is intended to reflect deviations from an individual’s baseline. This method offers a noninvasive and user-friendly approach suitable for real-life conditions and has been examined in previous research as a potential indicator for identifying menstrual cycle phases (Shilaih et al., 2017). The data was synchronized with the associated mobile application. A machine learning algorithm analyzed the assessed data to determine the phases of the menstrual cycle. Goodale et al. (2019) reported an accuracy of 90% in detecting fertile windows.

#### 2.3.4 Saliva hormone analyses

Saliva analyses were also included to verify menstrual cycle rhythms by measuring estradiol (E2) and progesterone (P). Saliva sampling provides a non-invasive and practical approach to assess menstrual cycle profiles (Gandara et al., 2007). This method has been validated for detecting irregular hormone fluctuations but not for identifying absolute hormone concentrations (Janse de Jonge et al., 2019). The saliva samples were either self-collected by the participants at home or collected by the principal investigators before an early morning practice session twice a week (usually on Tuesdays and Thursdays). The participants were instructed to follow a 10-h overnight fast and not to consume any liquids prior to sample collection. A small straw was used to collect the sample into a 10-mL tube, which was filled at least halfway with clear saliva. The samples were stored in either the home freezer or the research laboratory’s freezer at −20°C until the end of the study.

### 2.4 Data analysis

Data analysis was conducted using software R (version 4.2.2). Descriptive data were calculated for screening variables and monitored sleep parameters. Linear mixed-effects models (LMM) were assessed using the *lmer* function from the lme4 package (Bates et al., 2015). Each model included participant ID as a random intercept to account for repeated measurements. Model assumptions, including normality and homogeneity of residuals, were examined through diagnostic plots (Q-Q plots, residuals vs. fitted values, and histograms of residuals). Statistical significance was defined as *p* < .05, and 95% confidence intervals accompanied all estimates. Parameters were estimated using restricted maximum likelihood (REML).

Intraindividual (Level 1) parameters included menstruation status (yes/no) or menstrual cycle phase (early follicular, late follicular, luteal), along with continuous measures such as sRPE, number of perceived symptoms (0–18), and menstrual bleeding intensity. To account for intraindividual fluctuations and to isolate within-person effects, the continuous Level 1 variables were group-mean centered. This centering approach allows the interpretation of these predictors as deviations from each participant’s average. The between-person continuous Level 2 variables included age, the ASBQ score, and the MSi score. All Level 2 variables were collected within the initial psychometric screening. To facilitate interpretation and reduce potential multicollinearity with random effects, these continuous Level 2 predictors were grand-mean centered, allowing the fixed effects to be interpreted relative to the sample average. The dependent variables included sleep-related parameters and measures of perceived recovery and stress, represented by the eight items of the SRSS. Sleep parameters included sleep quality, restful sleep, total sleep time (TST), time in bed (TIB), sleep onset latency (SOL), and wake after sleep onset (WASO). These were calculated based on participants’ self-reported bedtimes and wake-up times. Time of lights out (TOL) and wake-up time (WUP) were combined with the corresponding date to generate full timestamps. SOL was added to TOL to determine the actual sleep onset time. TST was calculated as the duration between sleep onset and wake-up time, minus WASO. TIB was defined as the time between going to bed (TOL plus the time spent in bed before lights out) and waking up.

Violations of linear model assumptions, specifically non-normally distributed residuals and heteroscedasticity, were observed for the dependent variables sleep quality and Lack of Activation in models using the binary cycle classification, as well as SOL, WASO, and Mental Performance Capability in models based on AVA phase classification. To address these issues, the affected variables were log-transformed prior to model fitting. A natural logarithmic transformation was applied to reduce skewness and stabilize variance. This improved the residual distribution and enabled more reliable inference in the LMM. Consequently, the estimated effects for these outcomes are interpreted on the logarithmic scale. For clarity, model coefficients can be exponentiated and expressed as percentage changes in the original outcome variable.

Due to technical issues with data recording or synchronization of the Ava wristband in three participants, two different approaches were applied for the statistical analysis. In the first approach, the outcome variables were analyzed by differentiating the menstrual cycle into two phases using the binary variable *menstruation* (0 = *no menstruation*; 1 = *menstruation*) from the menstrual cycle diary as a predictor. This allowed for the inclusion of data from all eight participants, resulting in a total of 340 observations. While this approach did not permit phase-specific comparisons, it still enabled conclusions regarding other menstruation-related parameters. In a subsequent step, models with the same dependent variables were analyzed, replacing the binary variable *menstruation* with the categorical variable *cycle phase*. This predictor reflected individually algorithmically tracked cycle phases and included the categories *early follicular phase*, *late follicular phase*, *and luteal phase*, with the early follicular phase defined as the reference category. A total of 176 observations from five participants were included in this analysis. When a significant overall effect of cycle phase was identified, pairwise *post hoc* comparisons were conducted between all phases using Holm-adjusted *p*-values to control for the family-wise error rate.

The models for sleep quality, SOL, WASO, sleep efficiency, Physical Performance Capability, Overall Recovery, and Overall Stress, which included the Ava-based predictor, resulted in singular fits, where the estimated variance of the random intercept approached zero. This indicates that the between-subject variability in the outcome could not be reliably estimated in these cases, which may be due to the small number of participants in the subsample (n = 5) or limited variability across individuals. However, this does not imply that the random effect is irrelevant or that the model is equivalent to a fixed-effects model. To preserve the repeated-measures structure of the data and avoid violations of the independence assumption, the LMMs were retained. Fixed effects were interpreted with caution, considering the limitations associated with singular fits.

## 3 Results

A complete overview of all models is available in the Supplementary Material.

### 3.1 Descriptive statistics

A total of 8 out of 12 naturally menstruating female athletes, as defined by Elliot-Sale et al. (2021), completed the study. Two participants dropped out during the study: One after the first week and another after the second week. One participant did not complete the monitoring protocols and was therefore excluded. Another participant completed the study but reported the use of hormonal contraception, which led to her exclusion as well. In one case, the saliva samples collected in the second half of the study could not be analyzed due to errors during transportation. However, the remaining data of this participant were deemed sufficient for inclusion in the analysis. Descriptive statistics of the sleep parameters are provided in Table 2.

**TABLE 2:** Descriptive data of subjectively measured sleep parameters (n = 8).

	Mean ± SD	Min	Max	Median
Total Sleep Time, min	439.22 ± 90.86	95.00	785.00	410.00
Time in Bed, min	486.59 ± 92.19	170.00	960.00	465.00
Sleep Onset Latency, min	18.35 ± 25.61	0.00	180.00	10.00
Wake After Sleep Onset, min	8.82 ± 21.76	0.00	240.00	0.00
Sleep Efficacy, %	86.56 ± 10.81	47.80	100.00	90.40
Sleep Quality, 1-5	3.65 ± 1.17	1.00	5.00	3.00
Restful Sleep, 1-5	3.30 ± 0.95	1.00	5.00	3.00
Bedtime, hh:mm	00:13 ± 01:24	00:00	23:55	01:20
Wake Up, hh:mm	07:40 ± 01:32	03:00	12:50	08:00

Sleep Quality and Restful Sleep were examined using a 5-point Likert Scale, with higher values representing a higher state of quality or restfulness.

**FIGURE 2:**
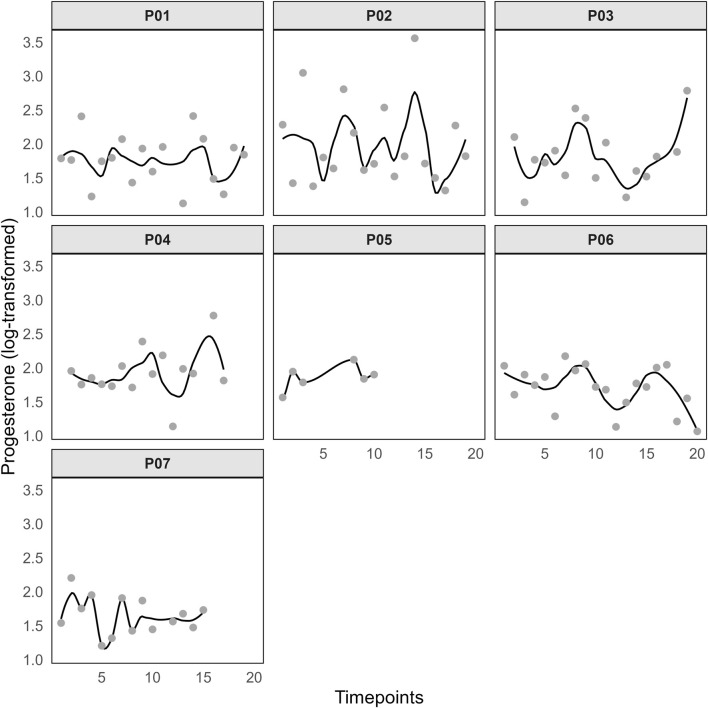
Log-transformed progesterone values. Notes: Timepoints represent the occasions of the saliva tests.

**FIGURE 3:**
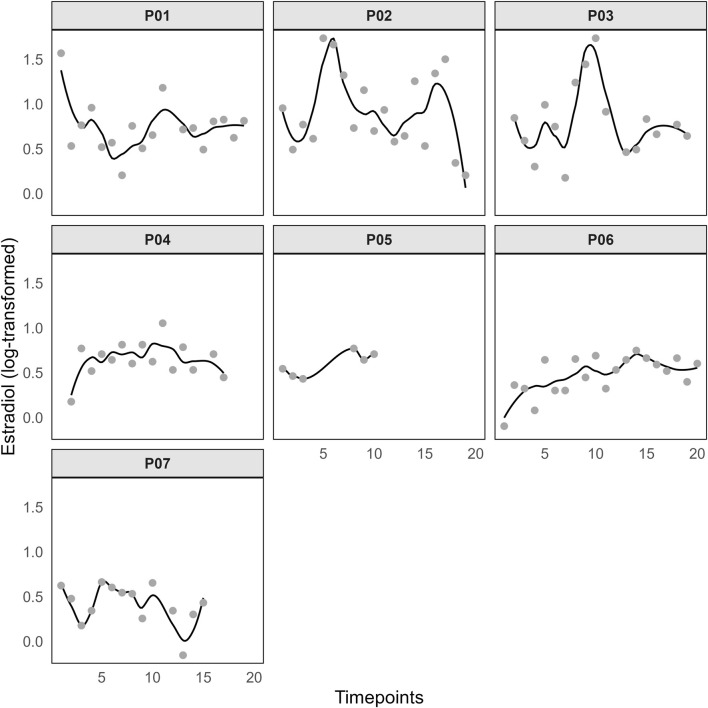
Log-transformed estradiol values. Notes: Timepoints represent the occasions of the saliva tests.

**FIGURE 4:**
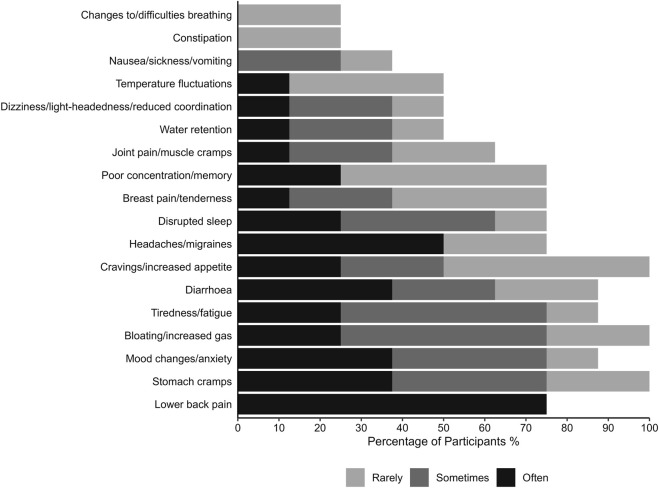
Frequency of menstrual cycle symptoms (measured by the Menstrual Symptom index; Bruinvels et al., 2021).

The average cycle length was 29.00 ± 1.20 days (Min = 28, Max = 31), with a median of 28.5 days, and the average menstruation duration was 5.75 ± 0.71 days (Min = 5, Max = 7), with a median of 6 days. The hormone curves of P (Figure 2) and E2 (Figure 3) are highly individual but indicate regular menstrual cycle patterns. However, the rise in progesterone levels indicates that ovulation has occurred, even if the exact timing cannot be determined precisely. The mean MSi score in this sample was 24.74 ± 12.53 (Min = 4, Max = 40). Additionally, all participants reported having a regular menstrual cycle during the psychometric screening. The distribution of symptom frequency is presented in Figure 4. ‘Cravings/increased appetite’, ‘bloating/increased gas’, and ‘stomach cramps’ were reported as the most common symptoms in this sample. Furthermore, participants were asked in which situations menstrual complaints affected them. In everyday life, four participants reported experiencing impairment often, three reported experiencing it rarely, and one reported no impairment. During training or competition, three participants reported impairments often, two reported no impairments, one reported them rarely, and two experienced impairments occasionally. Regarding sleep, three participants reported impairments frequently, two reported no impairments, two reported them rarely, and one reported experiencing impairments occasionally.

During the monitoring period, two players reported injury-related issues. One athlete was unable to participate in one training session due to an injury, practiced with limitations in eleven sessions, and was able to complete one session without restrictions during the injury phase. Despite the reported injury, the other player trained with limitations in two sessions and completed 13 sessions without restrictions.

### 3.2 Results for binary cycle classification

**FIGURE 5:**
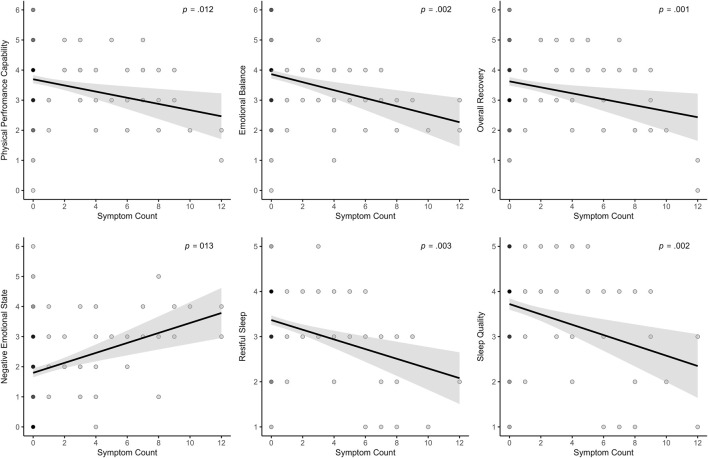
Effects of the daily measured number of symptoms on recovery, stress, and sleep parameters.

The analysis across all models revealed that symptom-related variables, sleep behavior, and menstrual complaints were consistently associated with several sleep and recovery-stress outcomes. A higher number of daily symptoms was significantly associated with poorer subjective sleep outcomes, including lower sleep quality (log-transformed; *β* = −0.03; *p* = .002), indicating that each additional symptom above an athlete’s individual average was linked to an approximate 3% decrease in sleep quality, and less restful sleep (*β* = −0.09; *p* = .003). Additionally, the perception of more symptoms was linked to lower Physical Performance Capability (*β* = −0.08; *p* = .012), lower states of Emotional Balance (*β* = −0.09; *p* = .002) and lower Overall Recovery (*β* = −0.10; *p* = .001). In the stress dimension, the number of symptoms was only positively associated with higher Negative Emotional State (*β* = 0.13; *p* < .001). All significant associations are visualized in Figure 5.

**FIGURE 6:**
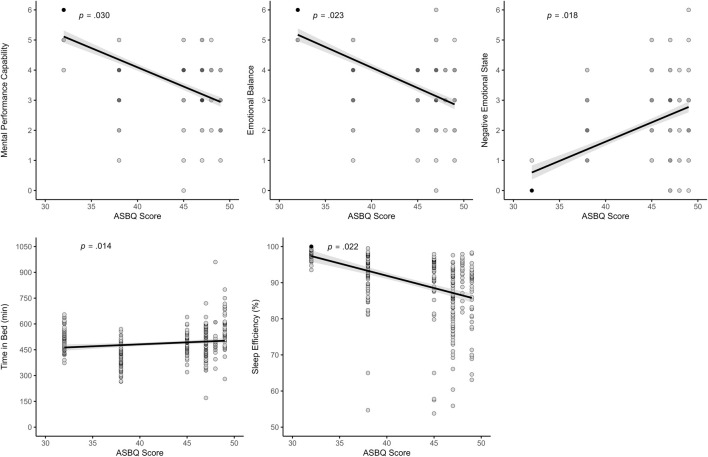
Effects of the ASBQ score on recovery, stress, and sleep parameters. Notes: ASBQ = Athlete Sleep Behavior Questionnaire.

The ASBQ score, with higher scores indicating less favorable sleep behavior, was negatively related to sleep efficiency (*β* = −0.76; *p* = .022). Higher ASBQ scores were also associated with more TIB (*β* = 7.16; *p* = .014). Regarding SRSS outcomes, more unfavorable sleep behavior was linked to lower Physical Performance Capability at trend level (*β* = −0.10; *p* = .065), lower Mental Performance Capacity (*β* = −0.12; *p* = .030), lower Emotional Balance (*β* = −0.12; *p* = .023), and higher Negative Emotional State (*β* = 0.09; *p* = .018). A trend-level effect was observed for higher Overall Stress (*β* = 0.06; *p* = .084). All significant associations are visualized in Figure 6.

**FIGURE 7:**
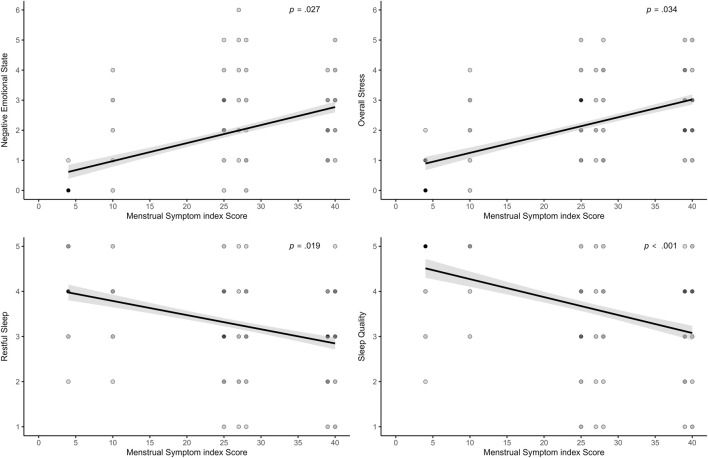
Effects of the MSi score on stress, and sleep parameters.

Higher MSi scores, reflecting more severe menstrual-related complaints, were associated with lower sleep quality (log-transformed; *β* = −0.009; *p* = .047), less restful sleep (*β* = −0.03; *p* = .019), and showed a trend-level association with more WASO (*β* = 0.39; *p* = .070). Regarding recovery-stress states, higher MSi scores were associated with lower Emotional Balance (*β* = −0.04; *p* = .083) at trend level, greater Negative Emotional State (*β* = 0.04; *p* = .027), and higher Overall Stress (*β* = 0.04; *p* = .034). All significant associations are visualized in Figure 7. However, menstrual bleeding intensity was only significantly associated with Overall Recovery, where higher values were linked to higher recovery scores (*β* = 0.17; *p* = .021). A trend-level negative association also appeared with Negative Emotional State (*β* = −0.14; *p* = .090). Higher sRPE was significantly related only to increased Muscular Stress (*β* = 0.03; *p* = .029), but no other associations were found. Finally, age was significantly associated with TIB, with older participants spending less time in bed (*β* = −8.72; *p* = .001). However, the binary menstruation variable did not yield a significant effect in any of the models.

### 3.3 Results for ava-based cycle classification

In the next step, the models for the previously analyzed outcome variables were re-estimated using algorithmically estimated cycle phase data collected via Ava wearables. Instead of the binary menstruation variable (yes/no) from the menstrual cycle diary, a categorical predictor representing the cycle phase (early follicular phase, late follicular phase, luteal phase) was included.

A higher number of daily symptoms was significantly associated with lower subjective sleep quality (*β* = −0.12; *p* = .018), less restful sleep (*β* = −0.10; *p* = .016), and longer WASO (log-transformed; *β* = 0.17; *p* = .013). Additionally, more symptoms were associated with lower scores on Overall Recovery (*β* = −0.14; *p* = .002) and higher scores on Negative Emotional State (*β* = 0.12; *p* = .013). However, the MSi score was associated with lower Physical Performance Capability (*β =* −0.23; *p* < .001) but also with lower Overall Stress (*β* = −0.14; *p* = .036). Regarding the sleep parameters, higher MSi scores predicted shorter SOL (log-transformed; *β* = −0.28; *p* < .001) and higher sleep efficiency (*β* = 1.31; *p* < .001). A similar effect was observed for the ASBQ score, which was also significantly associated with shorter SOL (log-transformed; *β* = −0.37; *p* < .001) and higher sleep efficiency (*β* = 1.52; *p* = .046). Moreover, higher ASBQ scores were associated with lower Physical Performance Capability (*β* = −0.28; *p* < .001) and lower Overall Stress ratings (*β* = −0.22; *p* = .021). Menstrual bleeding intensity was significantly related to higher ratings of Overall Recovery (*β* = 0.20; *p* = .033) and to lower ratings of Negative Emotional State (*β* = −0.29; *p* = .006).

**FIGURE 8:**
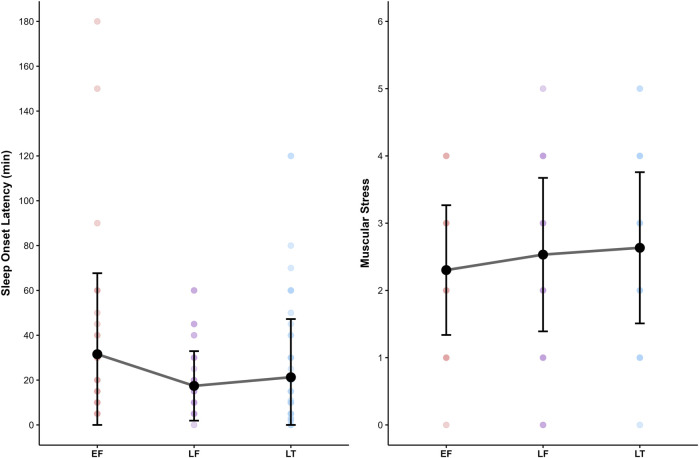
The effect of menstrual cycle phase on Sleep Onset Latency and Muscular Stress. Notes: EF, early follicular phase; LF, late follicular phase; LT, luteal phase. No *post hoc* comparisons reached significance despite a significant main effect of phase.

The menstrual cycle phase predictor was significantly associated with two outcomes. SOL (log-transformed) was shorter during the late follicular (*β* = −0.58; *p* = .037) and luteal (*β* = −0.60; *p* = .035) phases compared to the early follicular phase. Moreover, the luteal phase was significantly associated with higher values of Muscular Stress (*β* = 0.65; *p* = .035). However, for all outcomes, *post hoc* comparisons between cycle phases using the Holm method revealed no statistically significant pairwise differences (all *p* ≥ .10). The differences of the two outcome variables are visualized in Figure 8. Finally, age was significantly associated with higher sleep efficiency (*β* = 1.08; *p* < .001). No other outcomes showed a significant relationship with these covariates. No significant effects were found for the outcomes TST, TIB, Mental Performance Capability (log-transformed), Emotional Balance, and Lack of Activation.

## 4 Discussion

The present longitudinal study examined the influence of menstrual cycle phases and menstrual characteristics on sleep and recovery-stress states in elite basketball players. In addition to cycle phases, symptom-related variables (e.g., number of daily symptoms, bleeding intensity, and overall frequency, as measured by the MSi) as well as behavioral and demographic factors (e.g., sleep behavior, as measured by the ASBQ, training load, and age) were analyzed. Due to incomplete wearable data, the first approach used self-reported menstruation status (yes/no), and the second approach included algorithmically estimated cycle phases for a subsample. The findings indicate that individual symptom frequency and menstrual complaints exert a stronger and more consistent impact on sleep and recovery stress outcomes than menstrual cycle phases. These results highlight the importance of prioritizing individual menstrual experiences over generalized phase-based classifications when evaluating sleep and recovery dynamics in female athletes.

### 4.1 Discussion of results

Contrary to the initial hypothesis (H1), which suggested that athletes would demonstrate decreased sleep behavior during the luteal phase, the findings provide only limited support for phase-related effects on sleep. In the Ava-based models, the luteal phase was associated with a shorter sleep onset latency compared to the early follicular phase, even indicating a more favorable condition. However, *post hoc* comparisons between menstrual phases did not yield statistically significant pairwise differences. Additionally, no significant differences were found for other key sleep parameters. While the data of the present sample do not support phase-related effects on sleep, earlier research has suggested such effects may be present. Reduced subjective sleep quality during the luteal phase compared to the follicular phase has been observed in endurance athletes (De Martin Topranin et al., 2023) and Australian football athletes (Carmichael et al., 2021). However, effect sizes were small, and the authors concluded that menstrual cycle phase should be considered one of many potential stressors, rather than a primary driver of sleep alterations (De Martin Topranin et al., 2023). Although the effect on sleep quality was not confirmed in another recent study, objective sleep evaluation revealed longer wake after sleep onset, lower sleep efficiency, and more light sleep during the mid-luteal phase compared to the early follicular and ovulatory phases (Taylor et al., 2024). A further study using objective long-term monitoring revealed that sleep architecture varied modestly across menstrual cycle phases in junior endurance athletes (Hrozanova et al., 2021). The most distinct changes occurred during menstruation, with increased time spent in bed and deeper sleep, suggesting higher recovery needs. Moreover, recent findings in elite female rowers demonstrated that subjective sleep quality and well-being parameters (e.g., mood, energy levels, fatigue) fluctuate across menstrual cycle and hormonal contraceptive phases, supporting the general reliability and practical relevance of subjective measures in elite athlete monitoring (Antero et al., 2023). Taken together, the present findings do not align with previous suggestions. This may be partly explained by the reliance on subjective sleep measures only and the small sample size. Nevertheless, sleep diaries are widely used as a non-intrusive, cost-effective method that allows for long-term monitoring and provides valuable insights into athletes’ sleep-wake behavior. However, they are burdensome to complete, may be influenced by recall and social desirability biases, and typically overestimate sleep duration and efficiency compared to polysomnography (Halson, 2019; Walsh et al., 2021). Furthermore, it highlights the lack of a clear and unified direction in the existing literature on the effects of menstrual cycle phases on sleep.

In contrast to H1, the results related to H2 did not produce conflicting findings but offered only limited support for the hypothesized phase-related changes in recovery-stress states. Specifically, the luteal phase was associated with increased Muscular Stress in the Ava-based model. However, this effect was not confirmed in *post hoc* comparisons, and no other recovery or stress dimensions showed significant phase-related differences. Interestingly, a recent meta-analysis found that perceived muscular strain may vary across the menstrual cycle, using delayed onset muscle soreness (DOMS; Ohnhaus and Adler, 1975); as a measure of subjective muscular stress. Soreness levels were found to be highest in the early follicular phase and lowest in the mid-luteal phase, independent of the exercise protocol used (Romero-Parra et al., 2021). These differences were not reflected in objective markers such as creatine kinase, indicating a possible dissociation between perceived strain and physiological muscle damage. In contrast, Hackney et al. (2019) found that creatine kinase levels were significantly elevated following endurance exercise in the mid-follicular phase compared to the mid-luteal phase. Importantly, phase-related differences in recovery may also depend on the type of exercise performed (e.g., endurance vs. strength), making it even more complex to draw clear conclusions for sports such as basketball, where both types of physical demands are integrated within a single training or competition setting. More broadly, existing research provides no consistent evidence that recovery, mood, or perceived stress in athletes differ reliably between menstrual cycle phases. Several studies reported no significant variation in perceived recovery, fatigue, or mood across phases (Carmichael et al., 2021; Scott et al., 2024; Taylor et al., 2024). Some data indicate reduced physical readiness in the ovulatory and mid-luteal phases, while mental readiness appears unaffected (De Martin Topranin et al., 2023). Objective recovery indicators such as heart rate variability and inflammation have shown mixed results (Cabre et al., 2024). In addition, no consistent variation in physical or mental readiness based on bleeding status was noted, although a slight increase in resting heart rate was observed during the pre-bleeding days (Engseth et al., 2024).

Importantly, the binary menstruation variable (yes/no) did not show any effect in any of the calculated models, indicating that a methodological binary distinction of the menstrual cycle may not provide enough information and variance. However, the absence of statistically significant *post hoc* results should not be interpreted as evidence that no meaningful differences exist between menstrual cycle phases. Instead, this suggests that such effects may be subtle, highly individual, or masked by more dominant symptom-related influences. Moreover, a recent study comparing recovery-stress states in pre- and post-menarche athletes found more unfavorable stress responses in post-menarche athletes, indicating that multiple interacting factors may contribute to these differences (Kullik et al., 2025).

Across both modeling approaches, the number of daily reported symptoms showed consistent and robust associations with various sleep and recovery-stress outcomes. In the binary cycle classification model, a higher number of symptoms was significantly related to poorer subjective sleep outcomes, including reduced sleep quality and less restful sleep. Additionally, more symptoms were associated with lower Physical Performance Capacity, lower Emotional Balance, lower Overall Recovery, and an increased Negative Emotional State. In the Ava-based model, similar patterns emerged: symptom burden was again associated with lower sleep quality and restful sleep, along with increased wake after sleep onset. The findings provide strong support for both H3 and H4. H3 assumed that the association between menstrual symptoms and sleep quality would be evident, while H4 proposed that symptoms impair recovery-stress states. Taken together, the results highlight that daily symptom experience is a key predictor of both sleep and recovery-stress parameters. These findings align well with the growing body of literature emphasizing the importance of symptom burden over the menstrual phase. Several studies have reported that menstrual symptoms, rather than the cycle phase itself, are more closely associated with changes in sleep behavior and overall well-being. For instance, both Brown et al. (2025) and Halson et al. (2024) found that increased symptom frequency was associated with longer sleep durations and more frequent awakenings. In contrast, no consistent effects were observed for the menstrual phase alone. Beyond sleep, several studies provide compelling evidence that menstrual symptoms harm perceived performance, recovery, and psychological readiness. Increased symptom severity during menstruation has been linked not only to reduced exercise performance but also to delayed recovery perceptions following physical activity (McNulty et al., 2024b). This relationship appears particularly relevant in athletes who report multiple or intense symptoms such as pain, bloating, or fatigue. Supporting this, survey data from Olympic-level athletes showed that those experiencing three or more symptoms, menstrual pain, or regular use of analgesics were significantly more likely to perceive adverse performance outcomes, particularly during training phases (McNamara et al., 2022). Similarly, interviews with elite rugby players revealed that symptoms such as cramping, mood fluctuations, and fatigue were commonly perceived as impairing both physical and mental performance capacities (Findlay et al., 2020). Evidence from adolescent populations further supports this perspective. Many athletes reported symptom-related declines in training capacity and performance, especially during menstruation and the premenstrual phase, with fatigue, abdominal cramps, and lack of motivation among the most frequently reported complaints (Taim et al., 2023; Taim et al., 2024). These impairments often occurred in the absence of formal support structures. The daily number of symptoms and heaviness of bleeding were modeled as within-person predictors, allowing to capture of how deviations from each athlete’s own baseline influenced daily outcomes. Accordingly, increases above an individual’s average symptom level were associated with poorer sleep and recovery-stress states. This highlights the importance of individualized monitoring, as such deviations should be carefully considered in applied sport contexts.

In addition, cycle irregularities and heavy menstrual bleeding have been associated with shorter sleep duration, increased fatigue, and higher stress and depressive symptoms, suggesting a link between menstrual dysfunction and impaired recovery potential (Kennedy et al., 2022). However, across both modeling approaches, menstrual bleeding intensity was only weakly associated with sleep and recovery-stress outcomes. In the binary classification model, higher bleeding intensity was significantly linked to increased Overall Recovery and showed a trend-level negative association with Negative Emotional State, suggesting that individuals with heavier bleeding reported slightly more favorable recovery ratings. In the Ava-based model, similar associations were found: Menstrual bleeding intensity was significantly related to higher Overall Recovery and lower Negative Emotional State. One possible explanation for these findings is that athletes, in response, consciously or unconsciously adopt more active recovery strategies (e.g., increased rest and relaxation techniques) to manage physical stress. In this sense, the more favorable recovery ratings may reflect compensatory behaviors rather than a direct physiological benefit associated with the intensity of bleeding. In the present sample, no participants reported irregular menstrual cycles. Cycle regularity was verified through salivary hormone samples, ensuring hormonal profiling. Nonetheless, menstrual regularity remains an important factor to consider in the broader context of recovery and athlete health. Irregular cycles may reflect underlying hormonal imbalances or energy availability issues, both of which can influence recovery processes. Similarly, previous research has shown that athletes with irregular menstrual cycles are at greater risk for non-contact ankle and knee injuries, suggesting a link between menstrual regularity, motor control, and injury susceptibility (Vico-Moreno et al., 2022).

While daily symptom fluctuations on the intraindividual level showed consistent associations with sleep and recovery-stress outcomes, the results also indicate that inter-individual differences play a role, particularly for general menstrual symptom frequency and sport-specific sleep behavior. Across both modeling approaches, higher scores on the MSi were associated with lower subjective sleep quality and less restful sleep. In contrast, the Ava-based model revealed controversial findings: higher MSi scores were linked to shorter subjectively reported sleep onset latency and increased sleep efficiency, indicating more favorable sleep. Similarly, for the ASBQ, higher scores were associated with more unfavorable sleep behavior, lower sleep efficiency, and significantly more time in bed, but only in the binary model. In the Ava-based model, a higher ASBQ score was also linked to shorter sleep latency and higher sleep efficiency, but no other sleep outcomes showed significant associations. Aside from these associations with sleep latency and efficiency, which deviate from the expected direction, the only effect on further sleep parameters observed was the association between ASBQ and increased time in bed in the binary model. However, it is important to note that all sleep parameters were assessed subjectively, which may limit the accuracy of variables such as sleep latency and sleep efficiency. These metrics are often difficult to estimate without the use of objective tools, such as actigraphy. In contrast, the psychometric ratings of sleep, represented by sleep quality and restful sleep, showed consistent and theory-aligned associations with both MSi and ASBQ. This supports the utility of validated, multidimensional instruments in capturing meaningful variation in sleep-related recovery, highlighting the importance of considering both individual symptom burden and habitual sleep behavior when evaluating recovery processes.

Taken together, these findings suggest that psychoeducation and behavior-oriented strategies to improve sleep hygiene and routines are practical tools for enhancing recovery in athletes, particularly those with sport-specific sleep vulnerabilities. Interventions such as structured sleep education programs (Dunican et al., 2021) or brief individualized strategies (Gooderick et al., 2025) have shown clear benefits, highlighting the potential of both team-wide and low-threshold approaches. The consistent effects of MSi score and daily symptom burden highlight the need to move beyond a purely phase-based view when examining the menstrual cycle. The findings support the implementation of general menstrual health screenings in athletic settings as a basis for individualized recovery strategies. Previous work shows that regular cycle tracking and screenings are linked to fewer menstrual disorders (Kirschbaum et al., 2025), while qualitative studies underline persistent barriers such as stigma, lack of coach education, and limited institutional support (Jørgensen et al., 2025; Chica-Latorre et al., 2025a; Chica-Latorre et al., 2025b; Meijen and Martin, 2024). Yet even brief educational interventions have proven effective (Larsen et al., 2022), and staff acknowledged the value of menstrual tracking despite practical challenges (Carmichael et al., 2025). Broader efforts are still needed, as foundational education on female physiology remains limited (Hackney and Elliott-Sale, 2024). Finally, recent findings suggest that multifactorial approaches considering symptom profiles, circadian rhythms, and hormonal status (Seddik et al., 2025; McNulty et al., 2024b) may provide the most practical path forward. In addition, McNulty et al. (2024b) proposed a combined symptom frequency × severity score to better capture individual burden, which warrants further validation in relation to sleep and recovery. While daily hormonal sampling provides precision, it is rarely feasible in applied sport. The three-step method (Schaumberg et al., 2017) remains the gold standard, but its logistical demands limit use. A more practical alternative is the modified three-step method (Doroshuk and Doyle-Baker, 2025). Altogether, future studies should adopt longer-term, symptom-sensitive, and context-aware designs to advance individualized recovery strategies for female athletes.

### 4.2 Limitation

In addition to the important and novel findings, the present study has several limitations. A key aspect concerns the valid and detailed assessment of menstrual cycle phases. Although a combined approach using salivary hormone analyses and a fertility tracker was implemented, precise identification of individual cycle phases was not possible (Janse de Jonge et al., 2019). As only elite athletes were included, the more comprehensive three-method approach, consisting of calendar-based documentation, urine analysis, and blood analysis, could not be conducted due to restrictions imposed by the coaching staff and medical team. The Ava fertility tracker was used as a practical tool to assess cycle phases. Although previous studies have shown promising potential (e.g., Shilaih et al., 2017; Goodale et al., 2019), this study encountered significant challenges. Data collection was labor-intensive, and repeated device failures led to complete data loss in three cases. These limitations, along with inconsistent data quality, underline the risks of relying solely on wearables in applied sport settings (Elliott-Sale et al., 2025; Hunter and Smith, 2024). Nevertheless, the methods employed allowed for differentiation between anovulatory and ovulatory cycles, enabling at least a basic characterization of cycle patterns.

Another important limitation concerns the small sample size, which limits the statistical power. Consequently, only relatively large effects could be detected with confidence, whereas smaller but potentially meaningful associations may have gone unnoticed. Although twelve elite athletes were initially recruited, data from only eight participants could ultimately be included in the analysis. Still, the study yielded valuable initial insights suggesting that, in elite sports, individualized symptom monitoring and tailored support should take precedence over generalized recommendations. However, due to the relatively short study duration, only preliminary indications of individual profiles can currently be provided. Due to the relatively short study duration, only preliminary indications of individual profiles can currently be provided. Future research should therefore extend monitoring across entire seasons to capture long-term patterns, phase-specific effects, and the accumulated impact of symptoms under varying training and competition loads. This is particularly relevant during dense competition periods, such as playoffs, which place high demands on recovery. As shown by Conte et al. (2021), perceived fatigue and well-being can decline even when objective workloads remain stable, underscoring the importance of considering additional stressors such as hormonal fluctuations. Recent findings also emphasize the importance of moving beyond static phase-based models. Hormonal transitions, particularly from the luteal to the follicular phase, may be more physiologically demanding than the phases themselves and are often overlooked in traditional designs (Bruinvels et al., 2022). Continuous, individualized tracking could help capture these fluctuations more precisely.

A further limitation relates to the assessment of training load. Session RPE was reported on the following day, which may have introduced a potential memory bias compared to immediate post-session reporting. For organizational and practical reasons, however, this approach was the only feasible option within the study setting. Moreover, an important aspect not covered in the present study is the potential impact of hormonal contraceptive use. Based on existing knowledge, women who use hormonal contraceptives may exhibit different sleep patterns compared to non-users. However, this area remains insufficiently explored and should be addressed in future investigations.

## 5 Conclusion

The present study demonstrates that individual menstrual symptom frequency exerts a stronger and more consistent influence on sleep quality and recovery-stress states in elite basketball athletes than menstrual cycle phases alone. While phase-based effects were minimal, greater daily symptom burden was reliably linked to poorer sleep, reduced recovery, and increased stress. Sport-specific sleep behavior further contributed to these outcomes, underscoring the importance of healthy sleep routines. These findings highlight the need for individualized, symptom-focused approaches rather than generalized cycle-based models in athlete care. Integrating psychoeducational strategies focused on menstrual health and sleep hygiene into elite sport settings can empower athletes and enhance their self-management. Finally, implementing rigorous but practical menstrual tracking methods remains essential to ensure data quality and actionable insights. Overall, a multidimensional, athlete-centered framework that combines symptom monitoring, behavioral education, and robust cycle tracking is recommended to optimize the recovery and performance of female athletes.

## Data Availability

The raw data supporting the conclusions of this article will be made available by the authors, without undue reservation.
