# Correction: The impact of menstrual cycle phase and symptoms on sleep, recovery, and stress in elite female basketball athletes: a longitudinal study

**DOI:** 10.3389/fphys.2025.1713158

**Published:** 2025-10-14

**Authors:** Lisa Kullik, Eduard Isenmann, Jan Schalla, Michael Kellmann, Sarah Jakowski

**Affiliations:** ^1^ Department of Sport Psychology, Faculty of Sport Science, Ruhr University Bochum, Bochum, Germany; ^2^ Department of Fitness and Health, IST-University of Applied Sciences, Düsseldorf, Germany; ^3^ School of Human Movement and Nutrition Sciences, The University of Queensland, Saint Lucia, QLD, Australia

**Keywords:** menstrual cycle, sleep, recovery, basketball, elite athletes

Text correction

In the published article, the term **“severity”** was mistakenly used instead of **“frequency”**.

This has been corrected in multiple sections of the article, as shown below.

A correction has been made to the **Abstract**:

“In contrast, higher daily symptom burden and greater overall symptom frequency were consistently associated with poorer sleep quality, reduced recovery, and elevated stress. Additionally, sleep behavior significantly influenced both sleep and recovery outcomes.”

A correction has been made to the section **1 Introduction**:

“A higher frequency of menstrual symptoms was significantly associated with poorer sleep quality and more unfavorable sleep behaviors”.

A correction has been made to the section [**2.3.1 Psychometric screening**]:

“the Menstrual Symptom Index (MSi; Bruinvels et al., 2021), to assess symptom frequency systematically.”

A correction has been made to the section **3.3 Results for ava-based cycle classification**:

“(e.g., number of daily symptoms, bleeding intensity, and overall frequency, as measured by the MSi)”

“The findings indicate that individual symptom frequency and menstrual complaints exert a stronger and more consistent impact on sleep and recovery stress outcomes than menstrual cycle phases”.

A correction has been made to the section **4.1 Discussion of results**:

“For instance, both Brown et al. (2025) and Halson et al. (2024) found that increased symptom severity and frequency was associated with longer sleep durations and more frequent awakenings.”

“particularly for general menstrual symptom frequency and sport-specific sleep behavior.”

A correction has been made to the section **5 Conclusion**:

“The present study demonstrates that individual menstrual symptom frequency exerts a stronger and more consistent influence on sleep quality and recovery-stress states in elite basketball athletes than menstrual cycle phases alone.”

The original article has been updated.

