# Supplementary material for: The impact of menstrual cycle phase and symptoms on sleep, recovery, and stress in elite female basketball athletes: a longitudinal study
Source: Front Physiol. 2025 Sep 23;16:1663657. doi: 10.3389/fphys.2025.1663657 (PMC12500648; doi:10.3389/fphys.2025.1663657)
Supplement: Supplementary file 1 [file Table1.docx]

Supplementary Material

Supplementary Material A

Parameter set for the multilevel models with binary cycle classification.

| **Outcome** | ***B*** | ***SE*** | ***t*** | **(df)** | ***p*** |  |
| --- | --- | --- | --- | --- | --- | --- |
| **Sleep Quality** | | | | | | |
| Intercept | | 1.478 | 0.036 | 41.135 | (1, 4.734) | **< .001** |
| Age | | 0.006 | 0.008 | 0.726 | (1, 4.856) | .501 |
| ASBQ | | -0.009 | 0.006 | -1.372 | (1, 3.672) | .249 |
| MSi | | -0.009 | 0.003 | -2.789 | (1, 4.146) | **.047** |
| Menstruation | | 0.004 | 0.006 | 0.076 | (1, 339.00) | .939 |
| Heaviness of Bleeding | | 0.027 | 0.023 | 1.184 | (1, 338.70) | .237 |
| Number of Symptoms | | -0.003 | 0.009 | -3.194 | (1, 334.90) | **.002** |
| sRPE | | -0.001 | 0.003 | 0.244 | (1, 336.90) | .807 |
| **Restful Sleep** | | | | | | |
| Intercept | | 3.246 | 0.097 | 33.195 | (1, 5.717) | **< .001** |
| Age | | 0.018 | 0.023 | 0.816 | (1, 5.913) | .446 |
| ASBQ | | -0.024 | 0.017 | -1.435 | (1, 4.001) | .225 |
| MSi | | -0.032 | 0.009 | -3.486 | (1, 4.795) | **.019** |
| Menstruation | | -0.084 | 0.204 | -0.411 | (1, 338.511) | .681 |
| Heaviness of Bleeding | | 0.097 | 0.072 | 1.352 | (1, 338.981) | .177 |
| Number of Symptoms | | -0.087 | 0.029 | -2.969 | (1, 335.330) | **.003** |
| sRPE | | 0.002 | 0.009 | 0.248 | (1, 337.622) | .804 |
| **Total Sleep Time** | | | | | | |
| Intercept | | 449.789 | 13.366 | 33.651 | (1, 4.858) | **< .001** |
| Age | | -4.201 | 3.065 | -1.371 | (1, 4.912) | .230 |
| ASBQ | | 2.202 | 2.394 | 0.920 | (1, 4.106) | .409 |
| MSI | | -1.633 | 1.280 | -1.276 | (1, 4.441) | .265 |
| Menstruation | | -9.701 | 19.359 | -0.501 | (1, 331.270) | .617 |
| Heaviness of Bleeding | | -1.552 | 6.823 | -0.227 | (1, 330.951) | .820 |
| Number of Symptoms | | 0.022 | 2.779 | 0.008 | (1, 328.262) | .994 |
| sRPE | | -1.563 | 0.936 | -1.670 | (1, 329.873) | .096 |
| **Time in Bed** | | | | | | |
| Intercept | | 503.675 | 9.865 | 51.058 | (1, 5.434) | **< .001** |
| Age | | -8.718 | 2.268 | -3.844 | (1, 5.547) | **.001** |
| ASBQ | | 7.164 | 1.700 | 4.215 | (1, 3.915) | **.014** |
| MSI | | -0.438 | 0.926 | -0.473 | (1, 4.620) | .658 |
| Menstruation | | 3.652 | 19.315 | 0.189 | (1, 331.904) | .850 |
| Heaviness of Bleeding | | -3.375 | 6.816 | -0.495 | (1, 331.991) | .621 |
| Number of Symptoms | | 0.432 | 2.781 | 0.155 | (1, 328.261) | .877 |
| sRPE | | -0.882 | 0.936 | -0.942 | (1, 330.896) | .347 |
| **Sleep Onset Latency** | | | | | | |
| Intercept | | 17.931 | 3.011 | 5.956 | (1, 165.000) | **.001** |
| Age | | -0.240 | 0.693 | -0.035 | (1, 165.000) | .974 |
| ASBQ | | 0.809 | 0.522 | 1.548 | (1, 165.000) | .195 |
| MSI | | 0.442 | 0.284 | 1.558 | (1, 165.000) | .183 |
| Menstruation | | 4.954 | 5.719 | 0.866 | (1, 165.000) | .387 |
| Heaviness of Bleeding | | -0.927 | 2.018 | -0.459 | (1, 165.000) | .646 |
| Number of Symptoms | | 0.798 | 0.823 | 0.969 | (1, 165.000) | .333 |
| sRPE | | 0.315 | 0.276 | 1.139 | (1, 165.000) | .255 |
|  | |  |  |  |  |  |
| **Wake After Sleep Onset** | | | | | | |
| Intercept | | 8.428 | 1.940 | 4.344 | (1, 7.592) | **.003** |
| Age | | 0.240 | 0.449 | 0.535 | (1, 7.924) | .608 |
| ASBQ | | -0.200 | 0.312 | -0.641 | (1, 4.060) | .556 |
| MSi | | 0.391 | 0.177 | 2.207 | (1, 5.881) | .070 |
| Menstruation | | -0.822 | 4.977 | -0.165 | (1, 326.521) | .869 |
| Heaviness of Bleeding | | -1.462 | 1.759 | -0.832 | (1, 330.959) | .406 |
| Number of Symptoms | | 2.754 | 0.722 | 3.816 | (1, 330.746) | **<. 001** |
| sRPE | | -0.107 | 0.242 | -0.441 | (1, 333.714) | .659 |
| **Sleep Efficiency** | | | | | | |
| Intercept | | 89.334 | 1.210 | 73.848 | (1, 5.074) | **< .001** |
| Age | | 0.568 | 0.278 | 2.047 | (1, 5.137) | .095 |
| ASBQ | | -0.763 | 0.216 | -3.537 | (1, 4.205) | **.022** |
| MSi | | -0.218 | 0.112 | -1.891 | (1, 4.594) | .122 |
| Menstruation | | -1.523 | 1.845 | -0.826 | (1, 331.499) | .410 |
| Heaviness of Bleeding | | -0.003 | 0.651 | -0.004 | (1, 331.191) | .996 |
| Number of Symptoms | | -0.152 | 0.265 | -0.575 | (1, 328.371) | .566 |
| sRPE | | -0.115 | 0.089 | -1.291 | (1, 330.082) | .198 |
| **Physical Performance Capability** | | | | | | |
| Intercept | | 3.362 | 0.224 | 15.024 | (1, 4.317) | **< .001** |
| Age | | 0.026 | 0.051 | 0.513 | (1, 4.339) | .633 |
| ASBQ | | -0.103 | 0.041 | -2.511 | (1, 4.038) | .065 |
| MSi | | -0.031 | 0.022 | -1.447 | (1, 4.130) | .219 |
| Menstruation | | 0.169 | 0.215 | 0.786 | (1, 338.614) | .432 |
| Heaviness of Bleeding | | -0.003 | 0.076 | -0.041 | (1, 338.358) | .967 |
| Number of Symptoms | | -0.078 | 0.031 | -2.530 | (1, 337.089) | **.012** |
| sRPE | | 0.005 | 0.010 | 0.520 | (1, 337.681) | .604 |
| **Mental Performance Capability** | | | | | | |
| Intercept | | 3.517 | 0.198 | 17.768 | (1, 4.329) | **< .001** |
| Age | | 0.033 | 0.045 | 0.720 | (1, 4.352) | .509 |
| ASBQ | | -0.119 | 0.036 | -3.293 | (1, 4.029) | **.030** |
| MSi | | -0.039 | 0.019 | -2.042 | (1, 4.128) | .109 |
| Menstruation | | 0.134 | 0.197 | 0.681 | (1, 338.712) | .496 |
| Heaviness of Bleeding | | 0.001 | 0.070 | 0.014 | (1, 338.440) | .989 |
| Number of Symptoms | | -0.039 | 0.028 | -1.390 | (1, 337.085) | .166 |
| sRPE | | 0.004 | 0.009 | 0.465 | (1, 337.718) | .642 |
| **Emotional Balance** | | | | | | |
| Intercept | | 3.503 | 0.179 | 19.521 | (1, 4.360) | **< .001** |
| Age | | 0.024 | 0.041 | 0.583 | (1, 4.391) | .588 |
| ASBQ | | -0.119 | 0.033 | -3.638 | (1, 3.953) | **.023** |
| MSi | | -0.040 | 0.017 | -2.286 | (1, 4.089) | .083 |
| Menstruation | | 0.172 | 0.208 | 0.824 | (1, 339.199) | .410 |
| Heaviness of Bleeding | | -0.003 | 0.074 | -0.005 | (1, 338.851) | .996 |
| Number of Symptoms | | -0.092 | 0.030 | -3.085 | (1, 337.032) | **.002** |
| sRPE | | -0.002 | 0.010 | -0.156 | (1, 337.891) | .876 |
| **Overall Recovery** | | | | | | |
| Intercept | | 3.378 | 0.236 | 14.344 | (1, 4.370) | **< .001** |
| Age | | 0.021 | 0.054 | 0.395 | (1, 4.398) | .711 |
| Fsv | | -0.103 | 0.043 | -2.381 | (1, 4.120) | .074 |
| MSi | | -0.033 | 0.023 | -1.435 | (1, 4.224) | .221 |
| Menstruation | | -0.132 | 0.212 | -0.622 | (1, 336.540) | .535 |
| Heaviness of Bleeding | | 0.173 | 0.075 | 2.323 | (1, 336.316) | **.021** |
| Number of Symptoms | | -0.099 | 0.030 | -3.274 | (1, 335.159) | **.002** |
| sRPE | | -0.015 | 0.010 | -1.438 | (1, 335.667) | .151 |
|  | |  |  |  |  |  |
| **Muscular Stress** | | | | | | |
| Intercept | | 3.493 | 1.463 | 2.388 | (1, 0.973) | .258 |
| Age | | -0.043 | 0.102 | -0.425 | (1, 1.062) | .741 |
| ASBQ | | -0.178 | 0.344 | -0.517 | (1, 0.979) | .698 |
| MSi | | -0.166 | 0.248 | -0.670 | (1, 0.972) | .627 |
| Menstruation | | -0.255 | 0.368 | -0.691 | (1, 167.399) | .491 |
| Heaviness of Bleeding | | 0.032 | 0.122 | 0.265 | (1, 167.275) | .791 |
| Number of Symptoms | | -0.023 | 0.048 | -0.481 | (1, 167.002) | .631 |
| sRPE | | 0.039 | 0.020 | 1.992 | (1, 167.169) | **.048** |
| **Lack of Activation** | | | | | | |
| Intercept | | 0.896 | 1.094 | 8.186 | (1, 4.181) | **.001** |
| Age | | 0.012 | 0.025 | 0.488 | (1, 4.200) | .645 |
| ASBQ | | 0.034 | 0.020 | 1.693 | (1, 4.001) | .166 |
| MSi | | 0.013 | 0.011 | 1.254 | (1, 4.076) | .277 |
| Menstruation | | -0.045 | 0.085 | -0.523 | (1, 336.119) | .601 |
| Heaviness of Bleeding | | 0.019 | 0.030 | 0.632 | (1, 335.940) | .528 |
| Number of Symptoms | | 0.020 | 0.012 | 1.671 | (1, 335.033) | .096 |
| sRPE | | 0.004 | 0.004 | 0.988 | (1, 335.429) | .324 |
| **Negative Emotional State** | | | | | | |
| Intercept | | 2.038 | 0.133 | 15.245 | (1, 4.842) | **< .001** |
| Age | | -0.006 | 0.031 | -0.193 | (1, 4.968) | .854 |
| ASBQ | | 0.094 | 0.023 | 4.024 | (1, 3.751) | **.018** |
| MSi | | 0.042 | 0.013 | 3.320 | (1, 4.239) | **.027** |
| Menstruation | | 0.149 | 0.240 | 0.621 | (1, 338.985) | .535 |
| Heaviness of Bleeding | | -0.144 | 0.085 | -1.701 | (1, 338.743) | .090 |
| Number of Symptoms | | 0.126 | 0.035 | 3.646 | (1, 334.968) | **< .001** |
| sRPE | | 0.014 | 0.012 | 1.207 | (1, 336.929) | .228 |
| **Overall Stress** | | | | | | |
| Intercept | | 2.268 | 0.142 | 15.942 | (1, 5.369) | **< .001** |
| Age | | 0.023 | 0.033 | 0.710 | (1, 5.498) | .507 |
| ASBQ | | 0.056 | 0.025 | 2.244 | (1, 4.243) | .084 |
| MSi | | 0.040 | 0.013 | 2.945 | (1, 4.743) | **.034** |
| Menstruation | | -0.014 | 0.247 | -0.059 | (1, 338.930) | .953 |
| Heaviness of Bleeding | | -0.039 | 0.087 | -0.443 | (1, 338.649) | .658 |
| Number of Symptoms | | 0.012 | 0.036 | 0.329 | (1, 335.417) | .742 |
| sRPE | | 0.006 | 0.012 | 0.472 | (1, 337.055) | .637 |

*Notes: ASBQ = Athlete Sleep Behavior Questionnaire; MSi = Menstrual Symptom index; Menstruation (binary; 0 = no, 1 = yes); sRPE = Session Rating of Perceived Exertion.*

Supplementary Material B

Parameter set for the multilevel models with Ava-based classification.

| \| **Outcome** \| ***B*** \| ***SE*** \| ***t*** \| **(df)** \| ***p*** \| \| --- \| --- \| --- \| --- \| --- \| --- \| | | | | | |  |  |  |  |
| --- | --- | --- | --- | --- | --- | --- | --- | --- | --- | --- | --- | --- | --- | --- | --- |
| **Sleep Quality** | | | | | |  |  |  |  |
| Intercept | 3.315 | 0.425 | 8.042 | (1, 167.000) | **< .001** |  |  |  |  |
| Age | -0.053 | 0.033 | -1.590 | (1, 167.000) | .114 |  |  |  |  |
| ASBQ | -0.083 | 0.089 | -0.928 | (1, 167.000) | .355 |  |  |  |  |
| MSi | -0.043 | 0.063 | -0.678 | (1, 167.000) | .498 |  |  |  |  |
| Late follicular phase | 0.289 | 0.309 | 0.935 | (1, 167.000) | .351 |  |  |  |  |
| Luteal phase | 0.238 | 0.312 | 0.761 | (1, 167.000) | .448 |  |  |  |  |
| Heaviness of Bleeding | 0.135 | 0.102 | 1.328 | (1, 167.000) | .186 |  |  |  |  |
| Number of Symptoms | -0.116 | 0.049 | -2.380 | (1, 167.000) | **.018** |  |  |  |  |
| sRPE | -0.012 | 0.020 | -0.615 | (1, 167.000) | .540 |  |  |  |  |
| **Restful Sleep** | | | | | |  |  |  |  |
| Intercept | 3.980 | 0.753 | 5.287 | (1, 1.020) | .116 |  |  |  |  |
| Age | -0.019 | 0.053 | -0.356 | (1, 1.176) | .775 |  |  |  |  |
| ASBQ | -0.177 | 0.173 | -1.024 | (1, 0.925) | .503 |  |  |  |  |
| MSi | -0.146 | 0.124 | -1.176 | (1, 0.908) | .463 |  |  |  |  |
| Late follicular phase | 0.006 | 0.267 | 0.023 | (1, 166.808) | .982 |  |  |  |  |
| Luteal phase | -0.209 | 0.273 | -0.766 | (1, 166.875) | .445 |  |  |  |  |
| Heaviness of Bleeding | 0.026 | 0.088 | 0.296 | (1, 166.745) | .768 |  |  |  |  |
| Number of Symptoms | -0.102 | 0.042 | -2.427 | (1, 166.001) | **.016** |  |  |  |  |
| sRPE | -0.015 | 0.017 | -0.864 | (1, 166.747) | .389 |  |  |  |  |
| **Total Sleep Time** | | | | | |  |  |  |  |
| Intercept | 322.634 | 57.019 | 5.658 | (1, 1.014) | .109 |  |  |  |  |
| Age | -0.390 | 4.115 | -0.095 | (1, 1.280) | .937 |  |  |  |  |
| ASBQ | 22.905 | 12.840 | 1.784 | (1, 0.859) | .355 |  |  |  |  |
| MSi | 12.785 | 9.175 | 1.393 | (1, 0.830) | .429 |  |  |  |  |
| Late follicular phase | 39.467 | 26.882 | 1.468 | (1, 160.610) | .144 |  |  |  |  |
| Luteal phase | 20.574 | 27.236 | 0.755 | (1, 155.590) | .451 |  |  |  |  |
| Heaviness of Bleeding | 2.419 | 8.690 | 0.278 | (1, 161.842) | .781 |  |  |  |  |
| Number of Symptoms | 3.630 | 4.058 | 0.895 | (1, 161.020) | .372 |  |  |  |  |
| sRPE | 0.788 | 1.678 | 0.470 | (1, 161.995) | .639 |  |  |  |  |
| **Time in Bed** | | | | | |  |  |  |  |
| Intercept | 436.805 | 69.620 | 6.274 | (1, 1.015) | .098 |  |  |  |  |
| Age | -8.207 | 4.931 | -1.664 | (1, 1.182) | .315 |  |  |  |  |
| ASBQ | 18.331 | 4.909 | 1.152 | (1, 0.908) | .470 |  |  |  |  |
| MSi | 8.403 | 11.404 | 0.737 | (1, 0.887) | .608 |  |  |  |  |
| Late follicular phase | 24.079 | 26.754 | 0.900 | (1, 161.813) | .370 |  |  |  |  |
| Luteal phase | -1.903 | 27.146 | -0.070 | (1, 160.214) | .944 |  |  |  |  |
| Heaviness of Bleeding | 1.686 | 8.642 | 0.195 | (1, 161.993) | .846 |  |  |  |  |
| Number of Symptoms | 2.565 | 4.029 | 0.636 | (1, 161.013) | .525 |  |  |  |  |
| sRPE | 2.286 | 1.668 | 1.371 | (1, 161.913) | .172 |  |  |  |  |
| **Sleep Onset Latency** | | | | | |  |  |  |  |
| Intercept | 4.696 | 0.376 | 12.491 | (1, 164.000) | **< .001** |  |  |  |  |
| Age | -0.048 | 0.029 | -1.629 | (1, 164.000) | .105 |  |  |  |  |
| ASBQ | -0.367 | 0.079 | -4.627 | (1, 164.000) | **< .001** |  |  |  |  |
| MSI | -0.284 | 0.056 | -5.073 | (1, 164.000) | **< .001** |  |  |  |  |
| Late follicular phase | -0.585 | 0.278 | -2.101 | (1, 164.000) | **.037** |  |  |  |  |
| Luteal phase | -0.599 | 0.281 | -2.131 | (1, 164.000) | **.035** |  |  |  |  |
| Heaviness of Bleeding | -0.097 | 0.091 | -1.071 | (1, 164.000) | .286 |  |  |  |  |
| Number of Symptoms | 0.024 | 0.043 | 0.553 | (1, 164.000) | .581 |  |  |  |  |
| sRPE | 0.011 | 0.017 | 0.633 | (1, 164.000) | .527 |  |  |  |  |
|  |  |  |  |  |  |  |  |  |  |
| \| **Outcome** \| ***B*** \| ***SE*** \| ***t*** \| **(df)** \| ***p*** \| \| --- \| --- \| --- \| --- \| --- \| --- \| | | | | | |  |  |  |  |
| **Wake After Sleep Onset** | | | | | |  |  |  |  |
| Intercept | 1.378 | 0.600 | 2.299 | (1, 164.000) | **.023** |  |  |  |  |
| Age | 0.008 | 0.047 | 0.176 | (1, 164.000) | .861 |  |  |  |  |
| ASBQ | -0.128 | 0.126 | -1.017 | (1, 164.000) | .311 |  |  |  |  |
| MSi | -0.087 | 0.089 | -0.977 | (1, 164.000) | .330 |  |  |  |  |
| Late follicular phase | 0.382 | 0.444 | 0.861 | (1, 164.000) | .391 |  |  |  |  |
| Luteal phase | 0.202 | 0.448 | 0.450 | (1, 164.000) | .653 |  |  |  |  |
| Heaviness of Bleeding | -0.021 | 0.144 | -0.147 | (1, 164.000) | .884 |  |  |  |  |
| Number of Symptoms | 0.171 | 0.068 | 2.514 | (1, 164.000) | .**013** |  |  |  |  |
| sRPE | -0.031 | 0.028 | -1.132 | (1, 164.000) | .260 |  |  |  |  |
| **Sleep Efficiency** | | | | | |  |  |  |  |
| Intercept | 75.999 | 3.579 | 21.233 | (1, 162.000) | **< .001** |  |  |  |  |
| Age | 1.079 | 0.280 | 3.854 | (1, 162.000) | **< .001** |  |  |  |  |
| ASBQ | 1.521 | 0.756 | 2.013 | (1, 162.000) | .**046** |  |  |  |  |
| MSi | 1.308 | 0.533 | 2.454 | (1, 162.000) | **.015** |  |  |  |  |
| Late follicular phase | 2.650 | 2.631 | 1.007 | (1, 162.000) | .315 |  |  |  |  |
| Luteal phase | 3.822 | 2.648 | 1.443 | (1, 162.000) | .151 |  |  |  |  |
| Heaviness of Bleeding | -0.025 | 0.854 | -0.029 | (1, 162.000) | .977 |  |  |  |  |
| Number of Symptoms | 0.248 | 0.401 | 0.619 | (1, 162.000) | .537 |  |  |  |  |
| sRPE | -0.229 | 0.165 | -1.386 | (1, 162.000) | .168 |  |  |  |  |
| **Physical Performance Capability** | | | | | |  |  |  |  |
| Intercept | 3.919 | 0.372 | 10.541 | (1, 167.000) | **< .001** |  |  |  |  |
| Age | 0.057 | 0.029 | 1.940 | (1, 167.000) | .054 |  |  |  |  |
| ASBQ | -0.278 | 0.078 | -3.550 | (1, 167.000) | **< .001** |  |  |  |  |
| MSi | -0.226 | 0.055 | -4.083 | (1, 167.000) | **< .001** |  |  |  |  |
| Late follicular phase | 0.315 | 0.270 | 1.165 | (1, 167.000) | .246 |  |  |  |  |
| Luteal phase | 0.376 | 0.273 | 1.375 | (1, 167.000) | .171 |  |  |  |  |
| Heaviness of Bleeding | 0.065 | 0.089 | 0.734 | (1, 167.000) | .464 |  |  |  |  |
| Number of Symptoms | -0.071 | 0.043 | -1.673 | (1, 167.000) | .096 |  |  |  |  |
| sRPE | 0.005 | 0.017 | 0.263 | (1, 167.000) | .793 |  |  |  |  |
| **Mental Performance Capability** | | | | | |  |  |  |  |
| Intercept | 1.481 | 0.234 | 6.321 | (1, 1.013) | .098 |  |  |  |  |
| Age | 0.021 | 0.016 | 1.299 | (1, 1.100) | .403 |  |  |  |  |
| ASBQ | -0.044 | 0.054 | -0.811 | (1, 0.957) | .571 |  |  |  |  |
| MSi | -0.035 | 0.039 | -0.887 | (1, 0.947) | .544 |  |  |  |  |
| Late follicular phase | 0.076 | 0.063 | 1.197 | (1, 166.541) | .233 |  |  |  |  |
| Luteal phase | 0.117 | 0.065 | 1.813 | (1, 166.950) | .071 |  |  |  |  |
| Heaviness of Bleeding | 0.038 | 0.021 | 1.816 | (1, 166.4889 | .071 |  |  |  |  |
| Number of Symptoms | -0.015 | 0.010 | -1.490 | (1, 166.000) | .138 |  |  |  |  |
| sRPE | 0.002 | 0.004 | 0.428 | (1, 166.490) | .669 |  |  |  |  |
| **Emotional Balance** | | | | | |  |  |  |  |
| Intercept | 3.868 | 0.531 | 7.290 | (1, 1.029) | .082 |  |  |  |  |
| Age | 0.068 | 0.040 | 1.719 | (1, 1.484) | .269 |  |  |  |  |
| ASBQ | -0.213 | 0.117 | -1.822 | (1, 0.798) | .364 |  |  |  |  |
| MSi | -0.164 | 0.083 | -1.964 | (1, 0.765) | .354 |  |  |  |  |
| Late Follicular | 0.232 | 0.301 | 0.770 | (1, 166.777) | .442 |  |  |  |  |
| Luteal phase | 0.145 | 0.306 | 0.474 | (1, 160.644) | .636 |  |  |  |  |
| Heaviness of Bleeding | 0.039 | 0.099 | 0.391 | (1, 166.912) | .696 |  |  |  |  |
| Number of Symptoms | -0.075 | 0.047 | -1.585 | (1, 166.002) | .115 |  |  |  |  |
| sRPE | -0.006 | 0.019 | -0.294 | (1, 166.909) | .769 |  |  |  |  |
|  |  |  |  |  |  |  |  |  |  |
| \| **Outcome** \| ***B*** \| ***SE*** \| ***t*** \| **(df)** \| ***p*** \| \| --- \| --- \| --- \| --- \| --- \| --- \| | | | | | |  |  |  |  |
| **Overall Recovery** | | | | | |  |  |  |  |
| Intercept | 3.297 | 0.383 | 8.606 | (1, 167.000) | **< .001** |  |  |  |  |
| Age | 0.032 | 0.030 | 1.056 | (1, 167.000) | .293 |  |  |  |  |
| ASBQ | -0.139 | 0.081 | -1.717 | (1, 167.000) | .088 |  |  |  |  |
| MSi | -0.104 | 0.057 | -1.833 | (1, 167.000) | .069 |  |  |  |  |
| Late follicular phase | 0.191 | 0.279 | 0.685 | (1, 167.000) | .494 |  |  |  |  |
| Luteal phase | 0.103 | 0.282 | 0.365 | (1, 167.000) | .715 |  |  |  |  |
| Heaviness of Bleeding | 0.198 | 0.092 | 2.150 | (1, 167.000) | **.033** |  |  |  |  |
| Number of Symptoms | -0.137 | 0.044 | -3.122 | (1, 167.000) | **.002** |  |  |  |  |
| sRPE | -0.023 | 0.018 | -1.265 | (1, 167.000) | .207 |  |  |  |  |
| **Muscular Stress** | | | | | |  |  |  |  |
| Intercept | 3.024 | 1.612 | 1.876 | (1, 1.007) | .311 |  |  |  |  |
| Age | -0.044 | 0.111 | -0.395 | (1, 1.047) | .758 |  |  |  |  |
| ASBQ | -0.184 | 0.377 | -0.488 | (1, 0.980) | .712 |  |  |  |  |
| MSi | -0.172 | 0.271 | -0.633 | (1, 0.975) | .643 |  |  |  |  |
| Late follicular phase | 0.461 | 0.302 | 1.526 | (1, 166.283) | .129 |  |  |  |  |
| Luteal phase | 0.654 | 0.308 | 2.124 | (1, 166.601) | **.035** |  |  |  |  |
| Heaviness of Bleeding | 0.091 | 0.099 | 0.918 | (1, 166.252) | .360 |  |  |  |  |
| Number of Symptoms | -0.012 | 0.047 | -0.246 | (1, 166.000) | .806 |  |  |  |  |
| sRPE | 0.034 | 0.019 | 1.767 | (1, 166.253) | .079 |  |  |  |  |
| **Lack of Activation** | | | | | |  |  |  |  |
| Intercept | 3.190 | 1.942 | 1.643 | (1, 1.005) | .347 |  |  |  |  |
| Age | -0.067 | 0.134 | -0.500 | (1, 1.037) | .702 |  |  |  |  |
| ASBQ | -0.295 | 0.454 | -0.650 | (1, 0.984) | .635 |  |  |  |  |
| MSi | -0.212 | 0.327 | -0.647 | (1, 0.980) | .636 |  |  |  |  |
| Late follicular phase | -0.123 | 0.322 | -0.383 | (1, 166.226) | .702 |  |  |  |  |
| Luteal phase | 0.140 | 0.328 | 0.426 | (1, 166.493) | .671 |  |  |  |  |
| Heaviness of Bleeding | -0.040 | 0.106 | -0.376 | (1, 166.200) | .707 |  |  |  |  |
| Number of Symptoms | 0.089 | 0.050 | 1.763 | (1, 166.000) | .080 |  |  |  |  |
| sRPE | 0.020 | 0.021 | 0.981 | (1, 166.201) | .328 |  |  |  |  |
| **Negative Emotional State** | | | | | |  |  |  | (1, 166.253) |
| Intercept | 3.427 | 0.649 | 5.280 | (1, 1.029) | .114 |  |  |  |  |
| Age | -0.058 | 0.047 | -1.222 | (1, 1.339) | .395 |  |  |  |  |
| ASBQ | -0.156 | 0.146 | -1.073 | (1, 0.857) | .500 |  |  |  |  |
| MSi | -0.125 | 0.104 | -1.202 | (1, 0.830) | .471 |  |  |  |  |
| Late follicular phase | -0.562 | 0.314 | -1.792 | (1, 166.997) | .075 |  |  |  |  |
| Luteal phase | -0.497 | 0.319 | -1.557 | (1, 164.633) | .121 |  |  |  |  |
| Heaviness of Bleeding | -0.289 | 0.104 | -2.800 | (1, 166.995) | **.006** |  |  |  |  |
| Number of Symptoms | 0.124 | 0.049 | 2.517 | (1, 166.001) | **.013** |  |  |  |  |
| sRPE | 0.026 | 0.020 | 1.306 | (1, 166.995) | .193 |  |  |  |  |
| **Overall Stress** | | | | | |  |  |  |  |
| Intercept | 3.662 | 0.444 | 8.247 | (1, 167.000) | **< .001** |  |  |  |  |
| Age | -0.035 | 0.035 | -1.017 | (1, 167.000) | .311 |  |  |  |  |
| ASBQ | -0.217 | 0.093 | -2.325 | (1, 167.000) | .**021** |  |  |  |  |
| MSi | -0.140 | 0.066 | -2.119 | (1, 167.000) | **.036** |  |  |  |  |
| Late follicular phase | -0.232 | 0.323 | -0.718 | (1, 167.000) | .474 |  |  |  |  |
| Luteal phase | -0.297 | 0.327 | -0.910 | (1, 167.000) | .364 |  |  |  |  |
| Heaviness of Bleeding | -0.117 | 0.107 | -1.110 | (1, 167.000) | .273 |  |  |  |  |
| Number of Symptoms | < 0.001 | 0.051 | 0.003 | (1, 167.000) | .998 |  |  |  |  |
| sRPE | 0.013 | 0.021 | 0.628 | (1, 167.000) | .531 |  |  |  |  |

*Notes: ASBQ = Athlete Sleep Behavior Questionnaire; MSi = Menstrual Symptom index; sRPE = Session Rating of Perceived Exertion.*

Supplementary Material C

Post-hoc tests of significant effects of cycle phase.

| **Variable** | ***B*** | ***SE*** | ***t* ratio** | **(df)** | ***p*** |
| --- | --- | --- | --- | --- | --- |
| **Sleep Onset Latency** | | | | | |
| EF – LF | 0.585 | 0.278 | 2.101 | (1, 164) | .104 |
| EF – LT | 0.599 | 0.281 | 2.131 | (1, 164) | .104 |
| LF – LT | 0.014 | 0.157 | 0.088 | (1, 164) | .930 |
| **Muscular Stress** | | | | | |
| EF – LF | -0.461 | 0.302 | -1.524 | (1, 166) | .259 |
| EF – LT | -0.654 | 0.309 | -2.115 | (1, 167) | .108 |
| LF – LT | -0.194 | 0.175 | -1.105 | (1, 166) | .271 |
| **Negative Emotional State** | | | | | |
| EF – LF | 0.562 | 0.318 | 1.770 | (1, 167) | .236 |
| EF – LT | 0.496 | 0.329 | 1.511 | (1, 165) | .265 |
| LF – LT | -0.066 | 0.184 | -0.356 | (1, 167) | .722 |

*Notes: EF = early follicular phase; LF = late follicular phase; LT = luteal phase.*
